# Therapeutic potential of parkin as a tumor suppressor via transcriptional control of cyclins in glioblastoma cell and animal models

**DOI:** 10.7150/thno.57549

**Published:** 2021-11-01

**Authors:** Lila Rouland, Eric Duplan, Lígia Ramos dos Santos, Aurore Bernardin, Karen S. Katula, Guidalberto Manfioletti, Ahmed Idbaih, Frédéric Checler, Cristine Alves da Costa

**Affiliations:** 1 Université Côte d'Azur, INSERM, CNRS, IPMC, team labeled “Laboratory of Excellence (LABEX) Distalz”, 660 route des Lucioles, 06560, Sophia-Antipolis, Valbonne, France.; 2 Department of Biology, The University of North Carolina Greensboro, Greensboro, North Carolina, United States of America.; 3 Department of Life Sciences, University of Trieste, 34127 Trieste, Italy.; 4 AP-HP, Service de Neurologie 2 - Mazarin, Groupe Hospitalier Pitié-Salpêtrière, Université Pierre & Marie Curie Paris VI, Centre de Recherche de l'Institut du Cerveau et de la Moelle Epinière, UMRS 975, Paris, France.

**Keywords:** glioblastoma, parkin, cyclins, proliferation, transcription factor

## Abstract

Parkin (PK) is an E3-ligase harboring tumor suppressor properties that has been associated to various cancer types including glioblastoma (GBM). However, PK is also a transcription factor (TF), the contribution of which to GBM etiology remains to be established.

**Methods:** The impact of PK on GBM cells proliferation was analyzed by real-time impedance measurement and flow cytometry. Cyclins A and B proteins, promoter activities and mRNA levels were measured by western blot, luciferase assay and quantitative real-time PCR. Protein-protein and protein-promoter interactions were performed by co-immunoprecipitation and by ChIP approaches. The contribution of endogenous PK to tumor progression *in vivo* was performed by allografts of GL261 GBM cells in wild-type and PK knockout mice.

**Results:** We show that overexpressed and endogenous PK control GBM cells proliferation by modulating the S and G2/M phases of the cell cycle via the trans-repression of *cyclin* A and *cyclin B* genes. We establish that cyclin B is regulated by both E3-ligase and TF PK functions while cyclin A is exclusively regulated by PK TF function. PK invalidation leads to enhanced tumor progression in immunocompetent mice suggesting an impact of PK-dependent tumor environment to tumor development. We show that PK is secreted by neuronal cells and recaptured by tumor cells. Recaptured PK lowered cyclins levels and decreased GBM cells proliferation. Further, PK expression is decreased in human GBM biopsies and its expression is inversely correlated to both cyclins A and B expressions.

**Conclusion:** Our work demonstrates that PK tumor suppressor function contributes to the control of tumor by its cellular environment. It also shows a key role of PK TF function in GBM development via the control of cyclins *in vitro* and *in vivo*. It suggests that therapeutic strategies aimed at controlling PK shuttling to the nucleus may prove useful to treat GBM.

## Introduction

Glioblastoma multiforme (GBM) is the most aggressive brain tumor affecting adults. GBM usually signs a poor prognosis since the median survival time of patients does not overcome 15 months. It is characterized by infiltrative and invasive features, which preclude surgical full tumor resection and by consequence complete remission 1. GBM is usually classified according to the clinical presentation in primary/de novo and secondary GBM. Primary GBM is the most recurrent form and the analysis of the genetic alterations associated to its etiology indicates a strong correlation with abnormalities in signal transduction and cell cycle.

*PRKN* gene encodes for a protein named parkin (PK) that has been initially described as an ubiquitin ligase 2. *PRKN* is located at chromosome 6q25.2-27, a large genomic region that is frequently compromised in various cancers 3-6. Germ line *PRKN* mutations are associated to early-onset Parkinson's disease 7 while somatic *PRKN* loss of function mutations are linked to glioblastoma 8. Interestingly, we previously reported that PK expression was inversely correlated to the grade of brain tumors of different cell origins and that PK down-regulation in GBM resulted from a p53 loss of function 9. Several works have demonstrated that PK E3-ligase activity is associated to the development of brain tumors. Thus, it has been shown that PK overexpression in GBM cell lines triggers increased ubiquitination and degradation of cyclins D 10 and E 8 corroborating its implication as a master regulator of G1/S cyclins in various cancers 11. Overall, chromosomal localization, genetic mutations and cell biology data all concur to propose PK as a tumor suppressor.

We previously established that PK also behaves as a transcription factor 12, 13. Whether this novel function of PK also contributes to GBM setting and/or progression remained to be established. To address this question, we have examined PK ability to regulate GBM cells proliferation via the transcriptional control of key cell cycle players, namely cyclins A and B. We establish the respective contributions of PK E3-ligase function and TF on cyclins A and B regulations. E3 ligase function was assessed by blocking PK in the cytosol via its tagging with a NES (nuclear export signal) motif. PK controls the S and G2/M phases of the cell cycle via the transcriptional repression of cyclins A and B *in vitro* and *in vivo*. Interestingly, we show that the TF function of PK is implicated in the regulation of cyclins A and B while its E3-ligase activity participates to the control of cyclin B, only. Importantly, we show that neuronal secreted PK may control tumor development *in vitro* and *in vivo* and that its expression is correlated with the protein levels of cyclins A and B in human GBM biopsies.

## Methods

### Cellular and animal models

Human U87, 8MG and mouse GL261 glioblastoma naïve cell lines (kindly provided by Dr. H. Honnorat, Hospices Civils de Lyon), TSM1 neurons 14, SH-SY5Y and Mouse Embryonic Fibroblasts (MEF) were cultured in Dulbecco's Modified Eagle's Medium (DMEM) supplemented with fetal calf serum (10%), penicillin (100 U/mL) and streptomycin (50 µg/mL) and incubated at 37 °C in a 5% CO_2_ atmosphere. HAP1 is a haploid human cell line purchased from Horizon Genomics (HAP1 PK^+/+^) that was derived from KBM-7 cells (see 15). HAP1 clone 3208-5 (HAP1 PK^-/-^) was engineered using CRISPR-Cas9 and contains a frame shift mutation of PRKN. HAP1 cells were cultured in Iscove's Modified Dulbecco's Medium (IMDM) with 10% fetal calf serum, penicillin (100 U/mL) and streptomycin (50 µg/mL). Immortalized mouse embryonic fibroblasts invalidated for *PRKN* gene were kindly provided by Drs. T Dawson (Johns Hopkins University Hospital, Baltimore, MD, USA). Human cell lines were systematically validated by short-term tandem analysis (STR) method according to manufacturer's instructions (GenePrint® 10 System, Promega) and all models were tested for putative mycoplasma contamination by PCR according to 16. *Prkn* knockout mice have been provided by Dr. O. Corti (Institut du Cerveau et de la Moelle Epinière, Paris, France) and have been described 17.

U87, GL261 and TSM1 stably overexpressing wild-type PK were obtained by co-transfection of 1.9 μg of pSBbi and 100 ng of the pSB100X transposase coding vectors (CMV(CAT)T7-SB100) from Dr. Z. Izsvak, Addgene plasmid # 34879, 18) in 6‐well plates using the Lipofectamine™ 2000 transfection reagent according to the manufacturer's protocol (Thermo Fisher Scientific). Twenty‐four hours after transfection, cells were treated with puromycin (1 μg/mL) and selection was carried out for one week. Cells were trypsinized and polyclonal populations for empty vector (Mock) and wild-type PK were maintained in the presence of the corresponding selection drug.

In a subset of experiments, U87 stably overexpressing either the empty lentiviral vector FUW (Dr. D. Baltimore, Addgene plasmid # 14882, 19) or FUW vector containing wild-type PK, mutated (C431S) PK or NES-tagged PK were obtained by transduction approaches. Lentiviruses expressing these constructs were produced in the laboratory as described in 20.

### Human and mice sample preparations

Human samples were provided by the tumor bank of the Centre de Recherche de l'Institut du Cerveau et de la Moelle Epinière, Groupe Hospitalier Pitié-Salpêtrière UMRS 975, Paris, France. This cohort has been extensively described in 9. In this study, we have analyzed 11 grade IV (glioblastoma) and 10 control (non-related epilepsy surgery-derived) samples that have been extensively characterized regarding PK mutational status 9. As indicated in Viotti et al. 9, a written informed consent from all patients and validation by a local ethical committee were obtained for this study. The analysis was performed on anonymized data.

Human biopsies and control or PK knockout mouse brains of 6-10-month-old were introduced in MagNA Lyser Green beads tubes (Roche) and then homogenized for 45 s at 6,500 rpm in a MagNA Lyser instrument (Roche) in the appropriate buffer for protein and/or RNA extraction. For protein extraction, 1 mL of the protein extraction buffer (Tris-HCl 10 mM, pH 7.5 supplemented with a protease inhibitors cocktail (Sigma-Aldrich) and phosphatase inhibitors (sodium orthovanadate (1 mM), sodium fluoride (5 µM)) is added and the homogenates obtained were then submitted to sonication on ice before Western-blot analysis. For RNA extraction, 900 µl of RNA extraction lysis buffer (RNeasy® Plus Universal kit, Qiagen) was added and homogenates were submitted to mRNA extraction as previously described 21.

### Plasmid constructs and transfection approaches

*Cyclin A2* and *B1* promoters have been previously described 22, 23. The pGL2 vector containing a 213 bases fragment of human *Cyclin A2* promoter (described in 22) served as a template in a PCR reaction to generate the promoter deleted of the 5′-CTCCGGC-3′ nucleotides. This deleted motif (- 259 to - 253 from the start codon) corresponds to a putative PK binding site 13. The primers used were forward: 5'-CAAGAACAGCCGCCG GGGCTGCTCGCTGCA-3' and reverse 5'-TGCAGCGAGCAGCCCCGGCGG CTGTTCTTG-3'. The pGL2 vector: pCycB (- 287)-LUC (described in 23) containing a fragment of the human *Cyclin B1* promoter served as a template in a PCR reaction to obtain the promoter deleted of the 5'-CGGC-3' nucleotides corresponding to part of a putative PK binding site in position - 284 to - 278 from the start codon. The primers used were forward: 5'-CCTGGCCAGGCCTTCCTAGCCTCACTGTGGCCC-3' and reverse 5'-GGGCCACAGTGAGGCTAGGAAGGCCTGGCCAGG-3'. The eGFP-PKwt expressing vector, named pEGFP-PKWT, was a gift from Edward Fon (Addgene plasmid # 45875) and the peGFP-C2 corresponding empty vector comes from Clontech (Ref: 6083-1). CCNA2 (CCNCycA-Venus-Flag, addgene plasmid # 39853, a gift from Dr. J. Pines, 24) and CCNB1 (pcDNA 3/Cyclin B1, addgene plasmid # 39871, a gift from J. Pines, 25) containing vectors were obtained from addgene. The coding sequences of human CCNA2 and human CCNB1 were then PCR amplified using the following primers: forward: 5'-CCCAAGCTTCCACCATGTTGGGCAACTCTGCGCCG-3' and reverse: 5'-CGGGATCC TTACAGATTTAGTGTCTCTGGTGGGTTGAGG-3' for CCNA2 and forward: 5'-CCCAAGCTTCCACCATGGCGCTCCGAGCCACC-3' and reverse: 5'-CGGGATCCTTACACCTTTGCCACAGCCTTGGC-3' for CCNB1. The fragments were then digested with Hind III and Bam HI restriction enzymes and sub-cloned in the pcDNA 3.1 (+) vector (Invitrogen # V790-20) digested with the same enzymes. Wild-type human PK (PK^WT^) coding sequence (variant 1) already cloned in the pIRES2-EGFP-NEO vector (Clontech) 26 was amplified by PCR using the forward primer 5'-GTTAACCACCATGATAGTGTTT GTCAGGTTCAACTC-3' containing a HpaI restriction enzyme site (underlined) and the reverse primer 5'-GGCGCGCCTTACACGTCGAACCAGTGG-3' containing the AscI restriction enzyme site (underlined) and sub-cloned between the same restriction enzyme sites in the FUW lentiviral vector (already described in 19). We then used this FUW-PK^WT^ to generate the FUW-PK^C431S^ mutant (forward primer: 5'- AAAAATGGAGGCTCCATGCACATGAAG-3' and reverse primer: 5'-CTTCATGTGCATGGAGCCTCCATTTTT-3'). In order to obtain a N-terminal NES-tagged PK (PK^NESt^) in the FUW lentiviral vector, we designed and used the same strategy as for the cloning of PK^WT^ in FUW, but with the following primers: forward: (5'-GTTAACCACCATGCTCCTGGAACTGCTAGAAGACC-3') and reverse primer: (5'-GG CGCGCCTACACGTCGAACCAGTGGTC-3'). Other constructs were made in the pSB bicistronic vector: pSBbi-GP (Dr. E. Kowarz, addgene plasmid # 60511, 27). We inserted the wild-type human PK sequence in the pSBbi-GP vector. To do so, we digested the vector with SfiI enzyme and inserted the wild-type PK sequence previously amplified with the forward and reverse primers containing a SfiI site as described in 27 (forward primer: 5'-GGCCTCTGAGGCCACCATGATAGTGTTTGTCAGGTTCAACTCCAG-3' and reverse primer: 5'-GGCCTGACAGGCCCTACACGTCGAACCAGTGGTCCC-3'). The primers have been purchased from Eurogentec. The constructs were verified by full sequencing. Transient and stable transfections were carried out by means of Lipofectamine 2000 (Invitrogen) according to the manufacturer's instructions.

### Real time proliferation assay

The xCELLigence technology (ACEA Biosciences Inc.) allows the Real Time Cell Analysis (RTCA) of cell proliferation without chemical interference. The RTCA instrument was used to assess the proliferation of different cell models overexpressing or invalidated for PK. All the experiments have been done after a titration assay that allowed us to determine the optimal cell seeding density for each cell line. After an automatic scan of the plate to check for proper contact of the E-plate, the background impedance for each well of the plate was measured before the cells were plated and incubated for 30 min at 37 °C and 5% CO_2_ to allow their settling in an evenly distributed pattern. The E-plate 16 was then put in the cradle pocket of the RTCA apparatus and the data acquisition was initiated. The impedance signals were recorded every 5 min for the first 25 scans (2 h) and every 15 min until the end of the experiment (up to 72 h).

In a subset of experiments, in order to determine the impact of secretates on the proliferation of murine glioblastoma GL261, cells (40,000 per well) were plated on an E-plate 16. Two-three hours after seeding, cells were treated without changing the media with extemporaneously produced secretates (50 µg of proteins) of TSM1 EV or PKWT cells. The proliferation was recorded as described above.

### Co-culture experiments

For co-culture experiments, U87 naive cells (target cells corresponding to cells responding to stimuli and whose impedance will be measured) were plated (10,000 per well) on a E-plate 16. SH-SY5Y cells (effector cells producing the stimuli) over-expressing either PK or the empty bicistronic vector were plated (10,000 per well) in the E-plate Insert. The E-plate 16 containing the target cells is put immediately in the cradle of the apparatus to start the proliferation experiment while the E-plate insert containing the effector cells is incubated overnight before its assembly to the E plate 16, twenty-four hours after target cells seeding. The proliferation of target cell is continuously recorded for up to 150 hours according to the xCELLigence RTCA Co-Culture Device Protocol.

### Cell cycle analysis by Flow Cytometry

Cell cycle analysis was performed mostly as described 28 with a few modifications. U87 and MEF cells were grown in six-well plates with DMEM containing 10% serum. We used a partial deprivation of serum to stop the cell cycle at G1 phase. Cells were cultured at 37 °C for 24 hours in DMEM medium containing serum (0,1%) then returned to normal culture conditions (10% serum) for 24 hours to stimulate cell re-entry into the cell cycle. Cells were harvested, pelleted by centrifugation at 1,000 × *g* for 10 min at 4 °C, gently diluted in 500 μl of Phosphate-buffer Saline (PBS) containing propidium iodide (PI, 50 μg/mL) and RNAse A (1 mg/mL) and then incubated at 37 °C for 30 minutes. The PI fluorescence of individual nuclei was measured by using a FACScan flow cytometer (ACEA BIO, NovoCyte Flow Cytometer, 488 nm laser line, for excitation). Red fluorescence due to PI staining of DNA was expressed on a logarithmic scale simultaneously to the forward scatter of the particles. Fifty thousand events were counted on the scatter gate. All measurements were performed under identical conditions. Data analysis was performed by means of the NOVOEXPRESS^TM^ software.

### Cell fractionation

To obtain nuclear and cytosolic fractions, cells were harvested by centrifugation at 1,000 x g for 3 min at 4 °C, lysed and homogenized with a syringe (Agani Terumo 26 G needles) in 200 µl of HEPES buffer (20 mM, pH 7.4), containing 1.5 mM MgCl_2_, 1 mM EDTA, 1 mM EGTA, 1 mM DTT, protease inhibitors cocktail. Lysates were incubated in ice for 15 min then centrifuged at 850 × g for 5 min at 4 °C. Pellets (nuclear fractions) were homogenized in Tris buffer (50 mM, pH 7.4) containing 150 mM NaCl, 1 mM EDTA, 1% Triton X-100 (Sigma-Aldrich)], 0.5% deoxycholate (Sigma-Aldrich), 0.1% SDS supplemented with a protease inhibitors cocktail) and sonicated 2 × 15 s on ice. Lysates were centrifuged at 20,000 × g for 1 h 30 min at 4 °C to obtain a supernatant corresponding to the cytosolic fraction. Both fractions were submitted to Western blot analysis.

### Caspase-3 activity assay

Caspase-3 activity measurement was performed as extensively described 29.

### Immunoprecipitation of GFP-fusion proteins

The immunoprecipitation experiments were done with the Chromotek GFP-Trap Magnetic Agarose system according to the manufacturer protocol (Chromotek, Cat: gtm-20). An anti-Green Fluorescent Protein (GFP) Nanobody (VHH) covalently bound to magnetic agarose beads is used to immunoprecipitate control (eGFP protein expressed by the peGFP-C1 control vector) or GFP-PK fusion protein from U87 cell lysates. Briefly, U87 cells were plated at 450,000 cells per well of a 6 wells plate, transfected with peGFP-C1 vector empty or containing eGFP-Parkin construct in combination with either CCNA2, CCNB1 or the empty pcDNA 3.1 (+) vector. Twenty-four hours after transfection, cells were harvested and lysed in 200 µL of 10 mM Tris/HCL buffer pH 7.5 containing 150 mM NaCl, 0.5 mM EDTA, 0.5% Nonidet^TM^ P40 substitute and a protease inhibitors cocktail. Cell lysates were centrifuged at 17,000 × g for 10 min at 4 °C. Protein concentrations of supernatants were evaluated using the Bradford method and 100 µg of protein were incubated over night with pre-equilibrated beads according to the manufacturer protocol. After 4 washes with 500 µL of the above-described buffer, samples were resuspended in 2X SDS-sample buffer (Laemmli), boiled for 5 min at 95 °C, beads were separated with a magnet and the supernatants were analyzed along with the inputs (25 µg of total proteins per condition) by SDS-PAGE/Western blot.

### Secretates production

This protocol is adapted from Matafora & Bachi 30. TSM1 cells stably expressing EV or PK were plated in 5 × 15 cm dishes (5 × 10^6^ cells/dish). Twenty-four hours after seeding, the medium was discarded and attached cells were washed three times with 10 mL of autoclaved PBS and three times with serum-free medium. Cells were starved for 18 hours in 10 mL of serum-free DMEM. Media were collected and centrifuged at 250 × g for 5 min to eliminate dead cells. The supernatants were filtered with a 0.22 µm filters and the secreted proteins were concentrated by centrifugation (3200 × g) to a final volume of 1 mL using a microcon filter with 3 kDa cutoff (Millipore). To obtain a smaller final volume (around 200 µL), samples were further concentrated using microcon filters with 10 kDa cutoff (Millipore). The secreted protein concentrates were transferred into 1.5 mL Eppendorf tubes and sonicated (3 cycles: 30 s on/30 s off). Protein concentrations were analyzed with the Bradford method and samples were either used immediately for Western-blot analysis or to treat plated GL261 cells (200 µg/well of 6 well plates; 50 µg/well of X-CELLigence electronic microplates) or stored at -80 °C. In a subset of experiments, the dynamin inhibitor I, Dynasor, (Santa Cruz Biotechnology, sc-202592) was added for 20 hours at a final concentration of 40 µM prior to secretate treatment of GL261. For each secretate production, we verified that viability of the plated cells was over 95% using Trypan Blue stain and the Countess™ apparatus from Invitrogen™.

### Western-blot analysis

Samples (50-200 µg) from human biopsies, mouse brains and cell lines were loaded on 8% and 12% PROTEAN TGX stain-free gels and semi-dry transferred for 10 min by means of the precut blotting transfer pack (Biorad) and the Trans-Blot® Turbo™ Transfer System (pre-programmed Biorad protocol for 2 mini gels of 1.5 mm). Transferred proteins were immuno-blotted with anti-PK (Merck Millipore, MAB 5512), anti-cyclin A (Upstate biotechnology Cat: 06-138 Lot: 21306; Genetex, GTX 22097), anti-cyclin B1 (Genetex, GTX 22096, Millipore, Cat: 05-373 Lot: 2199734), anti-PCNA (Santa Cruz Biotechnology, sc-7907), anti-p53 (CM1 kindly provided by J.C. Bourdon, University of Dundee), anti-tubulin (Sigma-Aldrich, T 5168) and anti-actin (Sigma, clone AC-74, A 5316) antibodies. Immunological complexes were revealed with either anti-rabbit or anti-mouse IgG-coupled peroxidase antibodies (Jackson ImmunoResearch) by the electrochemiluminescence detection method (Roche Diagnostics S.A.S). Chemiluminescence was recorded using a luminescence image analyzer ChemiDoc™ Imaging System (Biorad) and quantifications of non-saturated images were performed with the Image lab 6.0.1 software. The utilization of stain-free imaging also allows normalization of bands to total protein in each lane.

### Promoter activity

Wild-type or mutated *Cyclin A* and *B* promoter activities were measured after co-transfection of 1 μg of the above described cDNAs and 1 μg of β-galactosidase cDNA in order to normalize the values obtained for transfection efficiencies as described 31. β-Galactosidase and luciferase activities were performed according to manufacturer's instructions by means of E 2000 and E 1500 kits (Promega), respectively.

### Protein-DNA interaction experiments

The interaction of endogenous PK protein with human *CCNA2* and *CCNB1* promoter regions harboring a putative PK-responsive element was examined in SH-SY5Y neuroblastomas cells by using the CUT&RUN Kit (Cell Signaling Technology, # 86652) according to the supplier's instructions. In brief, 10^5^ cells were harvested for each CUT&RUN reaction and input. Cells were immobilized with Concanavalin A Magnetic Beads, then treated with digitonin that favors the entry of primary antibodies and pAG-MNase fusion enzyme that cuts chromatin into fragments. The immunoprecipitation of PK was performed with the anti-PK antibody (Merck Millipore, MAB 5512). For positive and negative control reactions, we used anti-H3K4Me3 (mAb # 9751) and mAb IgG isotype, provided in the kit, respectively. The input samples were sonicated to yield 100-600 base pairs of chromatin fragments. DNA samples were purified and analyzed by qPCR. The design of primers for *CCNA2* and *CCNB1* promoter regions to be amplified took in consideration the PKRE localization sites identified *in silico* and validated by mutagenesis. For *CCNA2* promoter, primers used were: forward: 5'-GGTCCATTTCAATAGTCGCGGGAT-3' and reverse: 5'-AGCCAAAGACGCCCAGAGAT-3. For *CCNB1* promoter, primers used were: forward: 5'-CAGAGGCAGACCACGTGAGAG-3' and reverse: 5'-AGAATGCGTTTCCA GGGCGATC-3'. The Percent of Input (PI) was calculated using the formula PI= 100% * 2 (Ct 100% Input Sample - Ct IP Sample) where Ct is the average threshold cycle of qPCR. Results were expressed as percent relative to IgG (taken as 100%). All reaction samples signals were adjusted by Ct value of Spike-In DNA sample normalization.

### mRNA analysis in cells and mice brain

RNA were extracted and reverse transcribed as described 21 then samples were subjected to real-time PCR by means of a Rotor-Gene 6000 apparatus (Qiagen), using the SYBR Green detection protocol. Gene-specific primers designed with the Universal Probe Library System Assay Design software (Roche) were as follows: *cyclin A* (mouse forward: 5'-CTTGGCTGCACCAACAGTAA-3'; and reverse: 5'-CTTGGCTGCACCAACAGTAA-3'; human forward: 5'-CCATACCTCAAGTATTTGCCATC-3'; reverse: 5'- TCCAGTCTTTCGTATTAATGATTCAG-3'), *cyclin B* (mouse forward: 5'- GCGCTGAAAATTCTTGACAAC-3'; reverse: 5'-TTCTTAGCCAGGTGCTGCAT-3'; human forward: 5'-CATGGTGCACTTTCCTCCTT-3'; reverse: 5'- AGGTAATGTTGTAGAGTTGGTGTCC-3') and human *TP53* (forward: 5'-GAACCCTTGCTTGCAATAGG-3'; reverse: 5'-GTGAGGTAGGTGCAAATGCC-3'). The mRNA levels of these genes were normalized by means of the mouse *γ-actin* (forward: 5'-CACCATCGGTTGTTAGTTGCC-3'; reverse: 5'-CAGGTGTCGATGCAAACGTT-3') or human *GAPDH* (forward: 5'-TGGGCTACACTGAGCACCAG-3'; reverse: 5'-CAGCGTCAAAGGTGGAGGAG-3') mRNA expression levels.

### Immunofluorescence microscopy

U87 cells (100 000) transduced with Fuw vector or wild-type PK (PK^wt^) or GL261 cells (50 000) were plated on 18 mm coverslip previously coated with 0,05 mg/mL of L-poly-L-lysine for 2 hours at 37 °C. Then, cells were fixed with 4% paraformaldehyde for 30 min and permeabilized with 0,1 X Triton-X100 for 10 min. Nonspecific sites were blocked for 20 min with 5% BSA in PBS buffer containing 0,05% Tween-20. Each cover slip was incubated for 2 hours at room temperature with primary antibody (PK MAB 5512 Merck Millipore). A secondary donkey anti-mouse alexa fluor-488 antibody or donkey anti-mouse alexa fluor-594 antibody (Molecular Probes DMS, 1:1000) were used to reveal the primary antibody and the nuclei were stained using DAPI (Molecular probes, 1:15000). Images were acquired either using an inverted laser scanning confocal microscope Leica TCS SP8 3X (Leica Microsystems, Nanterre, France) through a HC PL APO CS2 100X/1.4 Oil immersion objective or with an epifluorescence microscope Axioplan2, Zeiss through a Plan-Apochromat 63×/1.40 oil DIC objective with immersion. Images were organized with OMERO, an open microscopy environment (OME). Final images were obtained after deconvolution using the Huygens Remote Manager version 3.7 (Scientific Volume Imaging, The Netherlands), using the CMLE algorithm, with SNR:45 and 40 iterations.

### Animal studies

C57BL6 control (PK^+/+^) and *PRKN* gene invalidated (PK^-/-^) mice were housed with a 12:12 h light/dark cycle and were given free access to food and water. All experimental procedures were in accordance with the European Communities Council Directive of 24 November 1986 (86/609/EEC) and local French legislation. 3-month-old males were used. GL261 cells (10^5^ cells in 4 μl) were intra-cranially injected in PK^-/-^ (n = 10) and wild-type littermates (n = 6) that were anesthetized with a mixture of ketamine (80 mg/kg) and xylazine (10 mg/kg). In a subset of experiments, PK^-/-^ mice groups (N = 29, males) aged of 3 months were injected with GL261 cells overexpressing an empty vector (n = 13), or wild type PK (n = 16). Tumor cells were implanted in the striatum with a 33-gauge needle at a depth of 3 mm, 1 mm lateral to midline and 1 mm anterior to bregma. Animals were monitored daily to assure welfare. Mice were euthanized 15 days following the implantation of tumor cells and their brains recovered for biochemical and tumor volume analysis.

### Tumor volume analysis

For each brain, serial coronal sections (30 μm) were obtained with a cryostat and directly mounted onto slides. Sections were fixed in ethanol 70° for 10 min at room temperature and then hematoxylin-eosin coloration was performed to visualize and locate the tumor. The number of sections with tumor (*n*) was counted and the height (*h*) and width (*w*) of the biggest tumor was measured. Tumor volume (mm^3^) was calculated according to the formula: *n* × 0.03 × *h × w*.

### Statistical analysis

Statistical analysis was performed with GraphPad Prism software (San Diego, California USA). The choice of parametric versus non-parametric test was established after assessment of the normality test (D'Agostino-Pearson omnibus Normality test) to assure Gaussian distribution of values. Two groups of variables that have passed the normality test were analyzed by unpaired Student's t-test while two groups of variables that have not passed the normality test were analyzed by the Mann-Whitney test. Analysis of more than two groups of variables that have passed the normality test was performed by ordinary One-way ANOVA while analysis of more than two groups of variables that have not passed the normality test were analyzed by Kruskal-Wallis test. Grouped analysis of one or more groups was performed by two-way ANOVA. All tests are two-sided; the mean was defined as the center value and errors bars correspond to SEM. The number of samples, replication of experiments, *post hoc* tests and the *p* values (stars) are provided in figures legends.

## Results

### PK controls GBM cells proliferation and cell cycle

**Figure 1:**
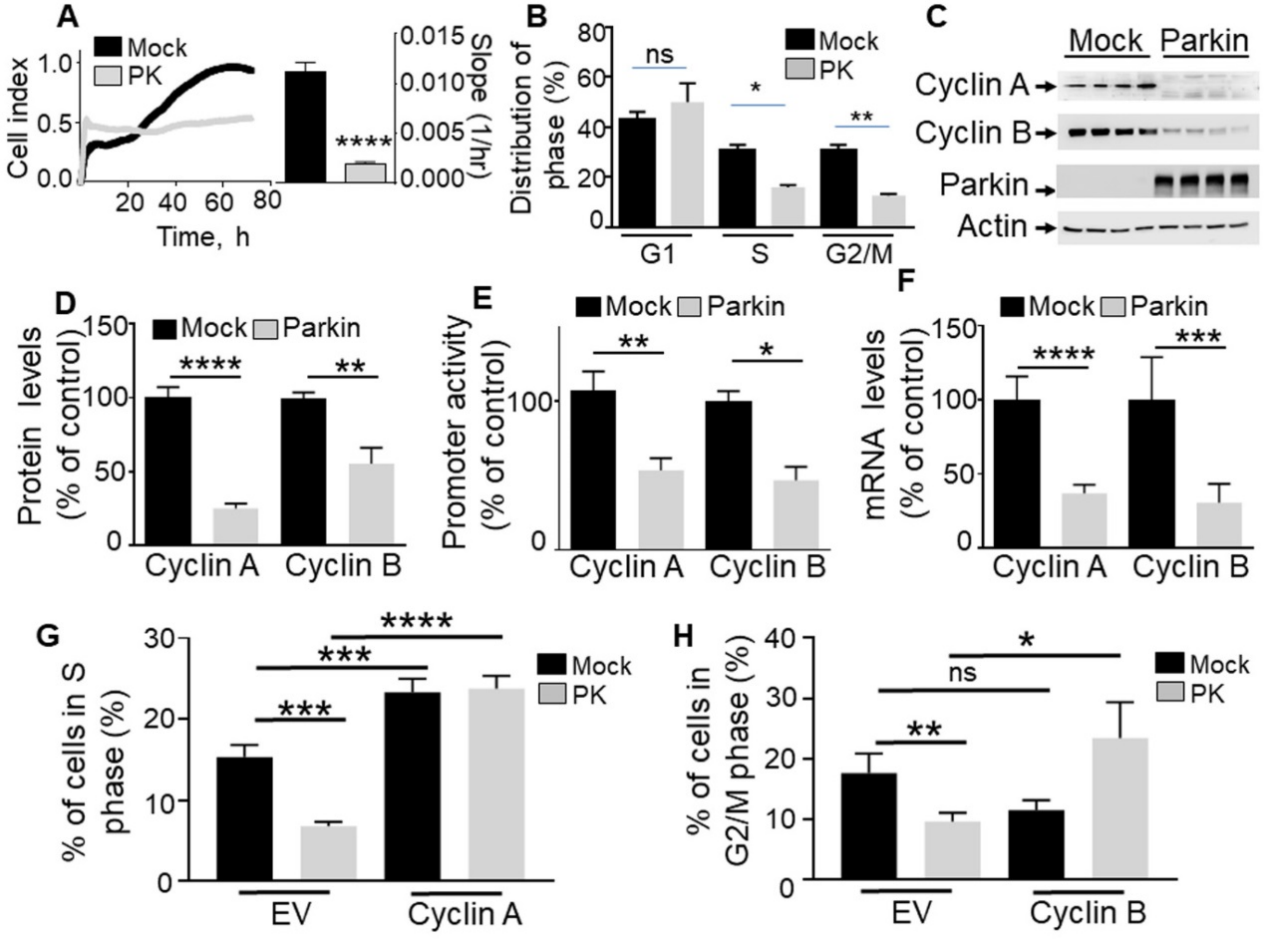
** PK controls proliferation in GBM cells via cyclins A and B regulation. (A, B)** Illustrate the effect of PK overexpression (by lentiviral approach) on cellular proliferation of U87 assessed by impedance-based label free approach (A, N = 9 Student's t test) and FACS (B, N = 6, Two-way ANOVA, Tukey's multiple comparison test) as described in Methods. Quantification analysis of the slopes of the curves are provided in A (right). Bars correspond to the means +/- SD (A) or SEM (B) of 2-3 independent experiments performed in triplicates. **(C-F)** U87 stably expressing an empty Fuw lentiviral vector (Mock, black bars) or wild-type PK (gray bars) were assessed for cyclins A and B protein expression (C, D, N = 9, analyzed by Student's t test), *cyclins A/B* promoter transactivation (E, N = 6, Mann-Whitney test) and *cyclins A/B* mRNA levels (F, N = 15, Mann-Whitney test) as described in Methods. Data are expressed as percent of Fuw control cells (taken as 100%) and are the means +/- SEM of 2-5 independent experiments performed in triplicates. Actin expression is provided in (C) as a control of protein load. * *p* < 0.05; ** *p* < 0.01; *** *p* < 0.001 and **** *p* < 0.0001. **(G, H)** Illustrate the effect of cyclin A (G, N = 9) and cyclin B (H, N = 9) over-expression in either control (Mock, black bars) or PK over-expressing U87 cells on the regulation of cell cycle phases by FACS as described in Methods. Bars correspond to the means +/- SEM (A) of 3 independent experiments performed in triplicates. Statistical analyses were performed by Two-way ANOVA, Tukey's multiple comparison test.

**Figure 2:**
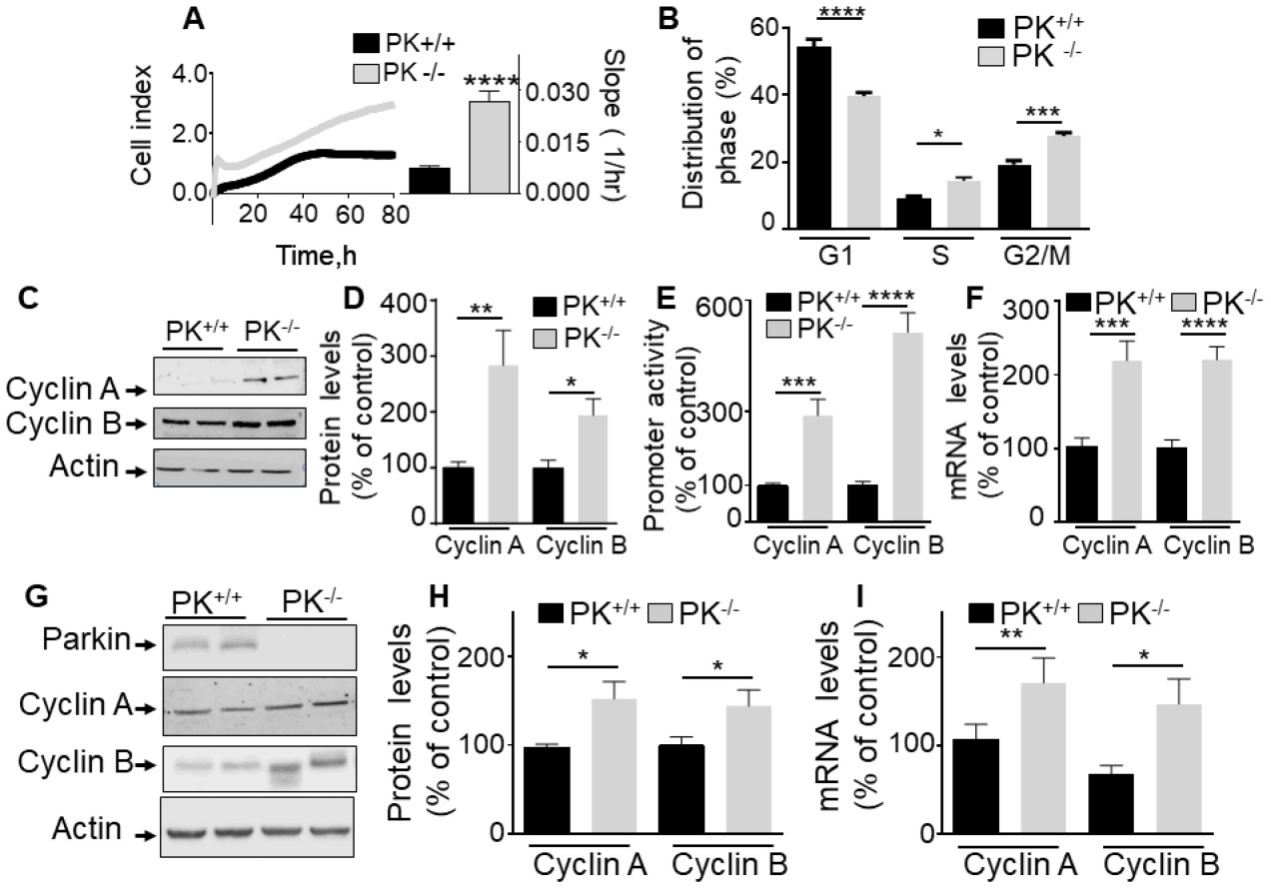
** Endogenous PK controls proliferation *in vitro* and in mice brain. (A, B)** Illustrate the effect of endogenous PK depletion on cellular proliferation of MEF cells assessed by impedance-based label free approach (A, N = 9 Student's t test) and FACS (B, N = 6, Two-way ANOVA, Tukey's multiple comparison test) as described in Methods. Quantification analysis of the slopes of the curves are provided in A (right). Bars correspond to the means +/- SD (A) or SEM (B) of 2-3 independent experiments performed in triplicates. **(C-F)** MEF control (PK^+/+^) or invalidated for PK (PK^-/-^) were assessed for cyclins A and B protein expression (C, D, N = 9, analyzed by Mann-Whitney test), *cyclins A/B* promoter transactivation (E, N = 15, Student's test) and *cyclins A/B* mRNA levels (F, N = 12, Student's test) as described in Methods. Data are expressed as percent of PK^+/+^ control cells (taken as 100%) and are the means +/- SEM of 2-5 independent experiments performed in triplicates. Actin expression is provided in (C) as a control of protein load. **(G-I)** PK^+/+^ and PK^-/-^ mice brain samples were examined for cyclins A and B protein expressions (G, H, N = 9, analyzed by Student's *t* test) and *cyclins A/B* mRNA levels (I, N = 9, Mann-Whitney test for cyclin A and Students' t test for cyclin B) as described in Methods. * *p* < 0.05; ** *p* < 0.01; *** *p* < 0.001; *p* < 0.0001.

To establish the contribution of PK in the control of cell cycle, we have analyzed the effect of the overexpression of PK on U87 proliferation rate and cell cycle phase distribution. Real-time assessment of the effect of wild-type PK overexpression shows a drastic reduction of cell proliferation as indicated by both cell index curves and their slopes (quantified during the exponential phase of proliferation, see histogram in **Figure 1A**). Corroborating this data, in MEFs, the depletion of endogenous PK (**Figure 2A**) yielded an opposite effect, i.e. an increase of cell proliferation and in slopes (**Figure 2A**). This set of data supports a tumor suppressor function of PK. Next, to determine whether an alteration of cell cycle distribution could account for the PK-linked modulation of cell proliferation, we have performed a flow cytometry analysis of both U87 and MEF cells labeled with propidium iodide, a classical DNA intercalator. U87 GBM cells overexpressing PK show a drastic reduction of the number of cells in phases S and G2/M but not G1 (**Figure 1B**) while, again, PK-depleted MEF cells display an increased number of cells in phases S and G2/M (**Figure 2B**).

### PK regulates *cyclins* A and B transcription in cells and mice brain

The fact that PK consistently impacted the S and G2/M cell cycle phases in cells either overexpressing or depleted of PK led us to investigate whether this could be accounted for by a control of cyclins A and B, two key modulators of S and G2/M phases respectively. **Figure 1C-F** clearly shows that PK overexpression in U87 cells (see expression in **Figure 1C**) triggers a strong reduction of cyclins A and B protein expressions (**Figure 1C, D**), promoter activity (**Figure 1E**) and mRNA levels (**Figure 1F**). This was not due to a putative artifact due to overexpression procedure since the depletion of endogenous PK in fibroblasts led to an opposite phenotype, i.e an increase of cyclins A and B protein expressions (**Figure 2C, D**), promoter activity (**Figure 2E**) and mRNA levels (**Figure 2F**). Of note, PK invalidation in human cells confirms an increase in cyclins A and B protein expressions (**Figure S1A**) and mRNA levels (**Figure S1B**). Of most importance, this set of cellular data was corroborated by the demonstration of an increase of cyclins A and B protein (**Figure 2G, H**) and mRNA levels (**Figure 2I**) in PK-invalidated mice brain. Overall, this set of data indicates that the control of cyclins by endogenous PK was not cell specific and occurs in cells as well as *in vivo*.

Next in order to address the contribution of either cyclin A and/or B in PK-mediated control of GBM cell proliferation, we have analyzed the impact of cyclins A and B rescue on the PK- mediated modulation of S and G2/M cell cycle phases, respectively. Our data show that cyclins A and B overexpression in both control and PK overexpressing U87 cells prevent PK-mediated S (**Figure 1G**) and G2/M (**Figure 1H**) phases reduction, respectively. This data highlights the key contribution of cyclins A and B in PK-mediated control of GBM cells proliferation.

### Identification and functional validation of a PK-responsive element on *cyclins A and B* promoters

**Figure 3:**
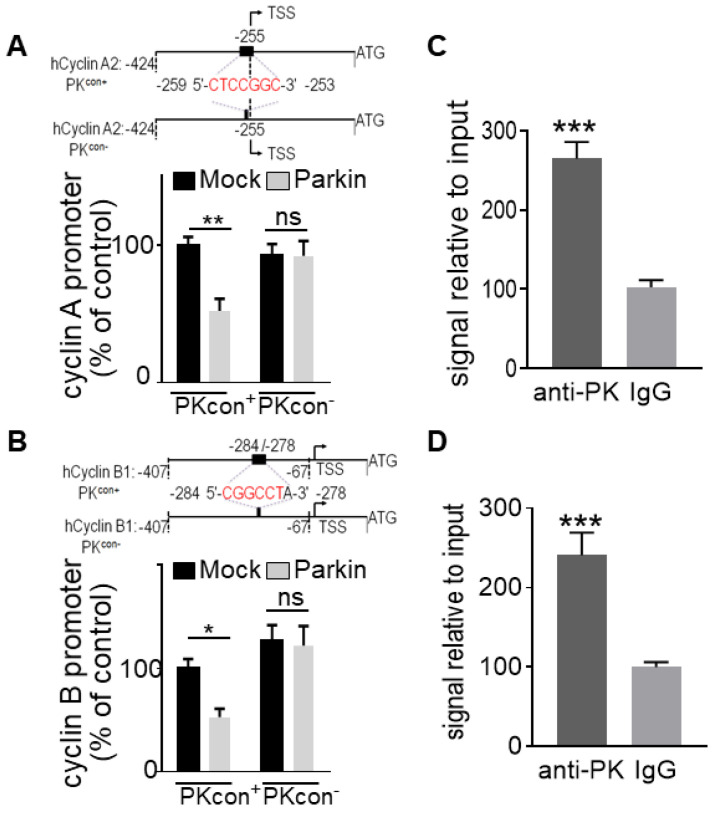
** Identification and validation of PK interacting domain on cyclins promoters. (A, B, upper panels)** Schemes represent the wild-type (PK^con+^) and the PK-responsive element-deleted (PK^con-^) human *cyclin A/B* promoters in frame with luciferase reporter. Black boxes correspond to putative PK responsive elements in each promoter. **(A, B, lower panels)** U87 cells stably over-expressing either empty Fuw vector (black bars) or PK (grey bars) were transfected with a mix of either cyclin A (A, N = 9, Two-way ANOVA, Tukey's multiple comparison test) or cyclin B (B, N = 9, Two-way ANOVA, Tukey's multiple comparison test) promoter and β-galactosidase reporter gene constructs in order to normalize transfection efficiencies and analyzed as described in Methods. Data are expressed as percent of Mock PK^con+^/β-galactosidase-transfected cells (taken as 100%) and are the means +/- SEM of 3 independent experiments performed in triplicates. **(C, D)** DNA-protein interaction of endogenous PK with PK-responsive element (PKRE) of *cyclin A2* (C, N = 6, Student's t-test) and *B1* promoter (D, N = 6 Student's t-test) regions in SH-SY5Y cells. Samples immunoprecipitated with antibodies directed against PK (anti-PK) show increased levels of cyclin A (C) and B (D) DNA than control (IgG) immunoprecipitated samples. Bars represent the means +/- SEM of 3 independent experiments in duplicates and are expressed as enrichment signal relative to input as described in Methods.

We have previously identified a “CGGCCT” sequence that we validated as a PK-responsive element (PKRE) 13. In this context, in order to support a putative contribution of PK TF function in PK-linked control of *cyclins A2* and *B1* genes, we first performed an *in silico* analysis of their promoters. Interestingly, both *cyclins A* (**Figure 3A**) and *B* (**Figure 3B**) promoters display a PKRE at positions - 259/- 253 and - 284/- 278, respectively. **Figures 3A** and **3B** confirmed that the overexpression of PK in U87 cells reduces the wild-type (PK^con+^) *cyclins A2* and *B1* promoter activities while PK failed to affect promoter activities of both *cyclins* promoters lacking the PKRE (compare (PK^con+^ and PK^con-^ in **Figure 3A, B**). Next in order to definitively demonstrate the implication of PK TF function in cyclins regulation, we have performed ChIP experiments. **Figure 3C, D** clearly shows that PK physically interacts with the promoter regions functionally validated in **Figure 3A, B**. Real-time PCR assessment indicates a physical interaction of PK with *cyclins A* (**Figure 3C**) and* B* (**Figure 3D**) promoters in SH-SY5Y. These data allowed us to definitely establish that PK regulates cyclins transcription directly, but this did not preclude a potential contribution of its E3-ligase.

### Contribution of PK E3-ligase activity to cyclins A and B regulation

**Figure 4:**
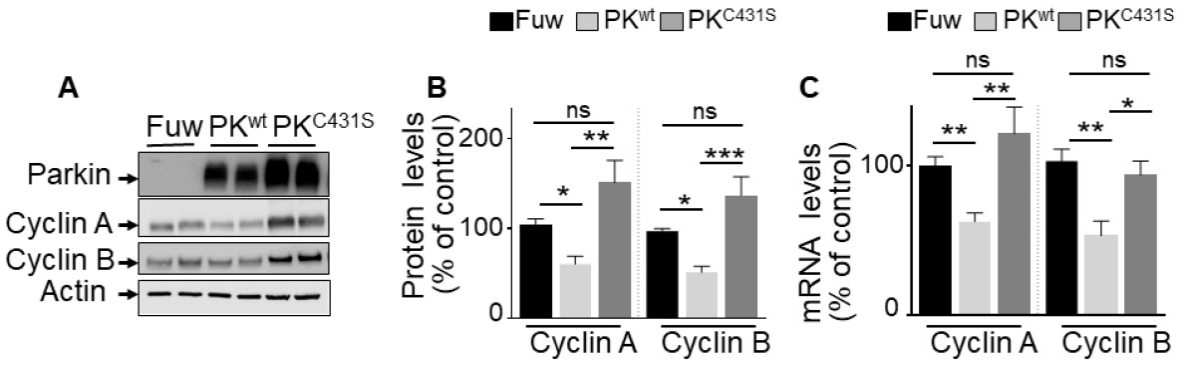
** Impact of PK E3-ligase activity on cyclins regulation in U87 GBM cells. (A-C)** U87 cells stably overexpressing an empty Fuw vector (black bars), wild-type PK (light grey bars) and mutated PK^C431S^ (dark grey bars) obtained by lentivirus approach were assessed for cyclins A and B protein expression (A, B, N = 9) and mRNA levels (C, N = 9) as described in Methods. Data are expressed as percent of Fuw control cells (taken as 100%) and are the means +/- SEM of 3 independent experiments performed in triplicates. Actin expression is provided in (A) as a control of protein load. Statistical analysis was performed by Kurskal-Wallis test/Dunn's multiple comparison test for cyclin A and ordinary one-way ANOVA/Tukey's multiple comparison test for cyclin B. * *p* < 0.05; ** *p* < 0.01; *** *p* < 0.001 and ns = non-significant.

Our promoter activities measurements and ChIP analysis did not preclude the possibility that, besides its TF function, PK-associated E3-ligase activity could also contribute to the control of cyclins A and B. Thus, we examined the impact of the C431S mutation of PK that is consensually considered as a key residue supporting its ligase activity 32. As expected, **Figure 4** shows that transient overexpression of wild-type PK (PK^wt^, **Figure 4A**) in U87 cells reduces cyclins A and B protein expressions (**Figure 4A, B**), a phenotype that was fully prevented by expressing the C431S mutation (**Figure 4A, B**). However, we observed that the C413S mutation also impacted the PK-mediated control of cyclins A and B mRNA levels (**Figure 4C**). Thus, the C431S mutation can also affect PK TF-mediated control of cyclins either directly by impairing PK TF function or indirectly via the control of an intermediate transcription factor controlling cyclins transcription and by consequence, whatever the case, also their protein expressions. We confirmed that as previously proposed 32 the C431S indeed abolishes PK-associated E3-ligase but concluded that this mutation could not be envisioned as a perfect tool to definitely discriminate between PK TF and E3-ligase-mediated activities.

### PK subcellular localization differentially impacts cyclin A and B regulation *in cellulo*

**Figure 5:**
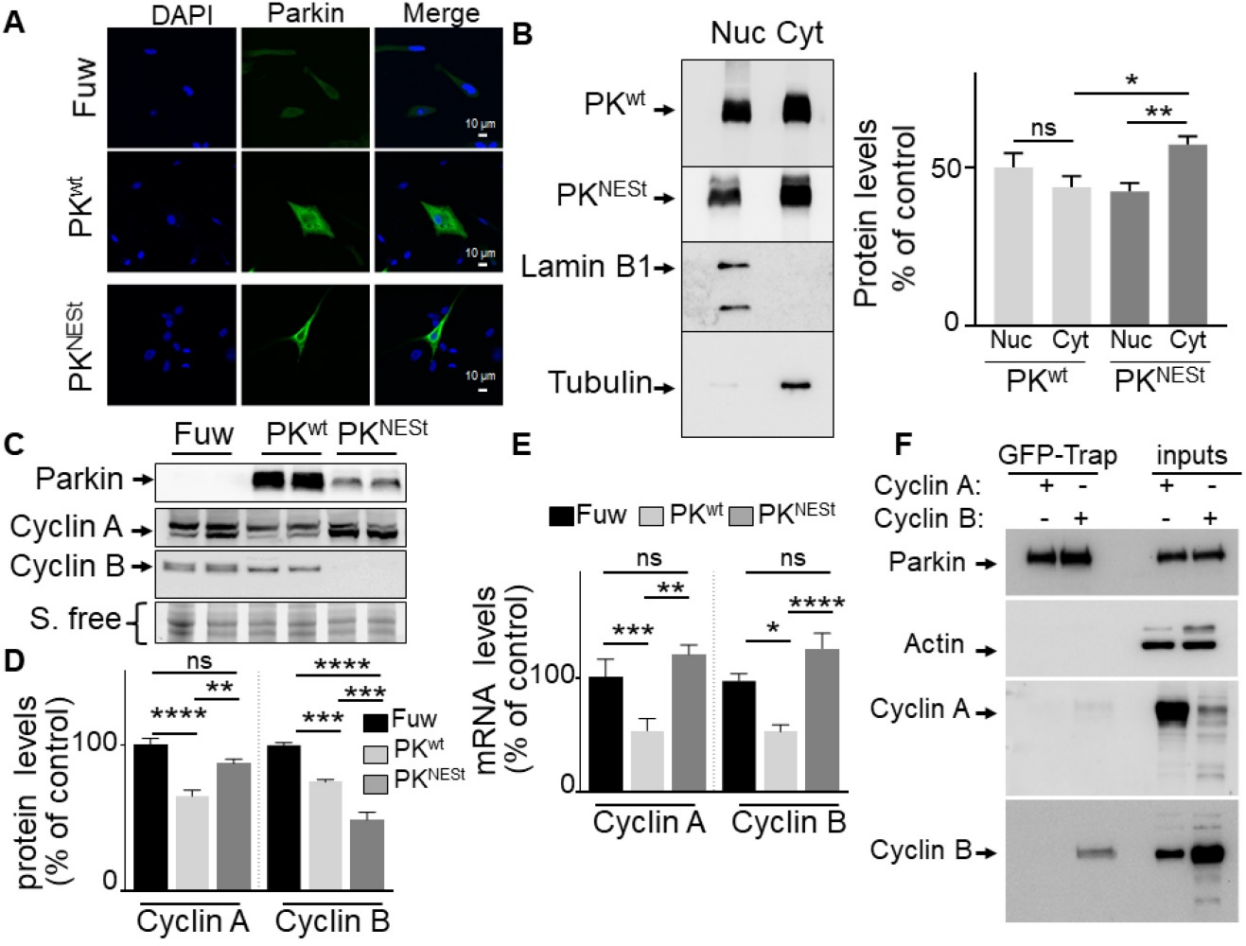
** Impact of cytosolic PK on cyclins regulation. (A)** PK subcellular localization was analyzed by immunofluorescence confocal microscopy in U87 stably expressing a lentivirus empty Fuw vector, wild-type PK (PK^wt^) and mutated PK (PK^NESt^) as described in methods. **(B)** PK subcellular localization analyzed by cell fractionation. PK^wt^, PK^NESt^, lamin B1 and tubulin immunoreactivities are shown. Bars represent PK expression in nuclear and cytoplasmic fractions and are expressed as percent of total (nuclear + cytoplasmic) immunoreactivities. The histogram represents the means +/- SEM of 3 independent experiments performed in duplicate. **(C, D)** The protein levels of cyclins A (N = 9, ordinary one-way- ANOVA/Tukey's multiple comparison test) and B (N = 6, ordinary one-way- ANOVA/Tukey's multiple comparison test) analyzed in the indicated cell types as described in Methods. **(E)** The mRNA levels of cyclins A (N = 6, Kruskal Wallis test/Dunn's test) and B (N = 9, ordinary one-way ANOVA/Tukey's multiple comparison test) were analyzed in the indicated cell types as described in Methods. Data are expressed as percent of Fuw control cells (taken as 100%) and are the means +/- SEM of 2-3 independent experiments performed in triplicates. Stain free labeling (S. Free) is provided in (A) as a control of protein load. * *p* < 0.05; ** *p* < 0.01; *** *p* < 0.001; **** *p* < 0.0001 and ns = non-significant. **(F)** U87 cells were transfected with peGFP-PKwt vector and the pcDNA 3.1 vector containing either CCNA2 (cyclin A2) or CCNB1 (cyclin B1) coding sequences. Twenty-four hours after transfection, cells were harvested, lyzed, and eGFP-PK was immunoprecipitated using the Chromotek GFP-Trap Magnetic Agarose system from Chromotek as described in Methods. PK, cyclin A and cyclin B expressions are from the same experiment. Three independent experiments have been done. Actin serves as a loading control for the inputs in which overexpressions of cyclin A or cyclin B are monitored. * *p* < 0.05; ** *p* < 0.01; *** *p* < 0.001 and ns = non-significant.

In order to overcome the limitations of the C431S PK and definitely delineate the contribution of PK ubiquitin-ligase function to cyclins regulation, we have generated a PK construct in which a nuclear export signal domain (NES) was N-terminally grafted to the wild-type PK sequence (PK^NESt^). First, in agreement with the dual function of PK, we confirm that wild-type PK partitioned between both nucleus and cytosol compartments while as expected, the PK^NESt^ is lower in the nuclear compartment and statistically significantly enriched in the cytosolic compartment (**Figure 5A, B**) as evidenced by both microscopy and subcellular fractioning approaches. Of note, Western-blot analysis shows that whole cellular PK^NESt^ expression was strikingly lower than that of wild-type PK, suggesting that PK^NESt^ was likely less stable in the cytosolic compartment (**Figure 5C**). Of importance, PK^NESt^ expression fully abolished wild-type PK-associated reduction of *cyclins A* and* B* mRNA levels (**Figure 5E**), indicating that the control of cyclins mRNA was indeed linked to the nuclear localization of PK and thus, genuinely related to PK TF function. Interestingly, PK^NESt^ significantly reduced cyclin B protein expression (**Figure 5C, D**). This can be likely explained by the fact that the enhancement of E3-ligase by cytosolic enrichment of PK^NESt^ further strengthened cyclin B ubiquitination and degradation. Conversely, unlike wild-type PK, PK^NESt^ expression did not affect cyclin A expression (**Figure 5C, D**). Overall, this data indicates that the reduction of cyclin A expression is linked to PK nuclear localization and due to its TF function. Overall, it can be concluded that cyclin A is only modulated by PK TF function while both ubiquitin-ligase and TF functions of PK participate in the control of cyclin B levels.

Interestingly, we have performed co-immunoprecipitations studies to evaluate the PK/cyclins protein physical interaction in U87 cells and we do confirm the interaction of PK with cyclin B (**Figure 5F**, lower panel), but not with cyclin A (**Figure 5F**). This data corroborates our conclusion that cyclin A is exclusively regulated by PK TF function while both E3-ligase and TF functions of PK are involved in cyclin B regulation in GBM cells.

**Figure 6:**
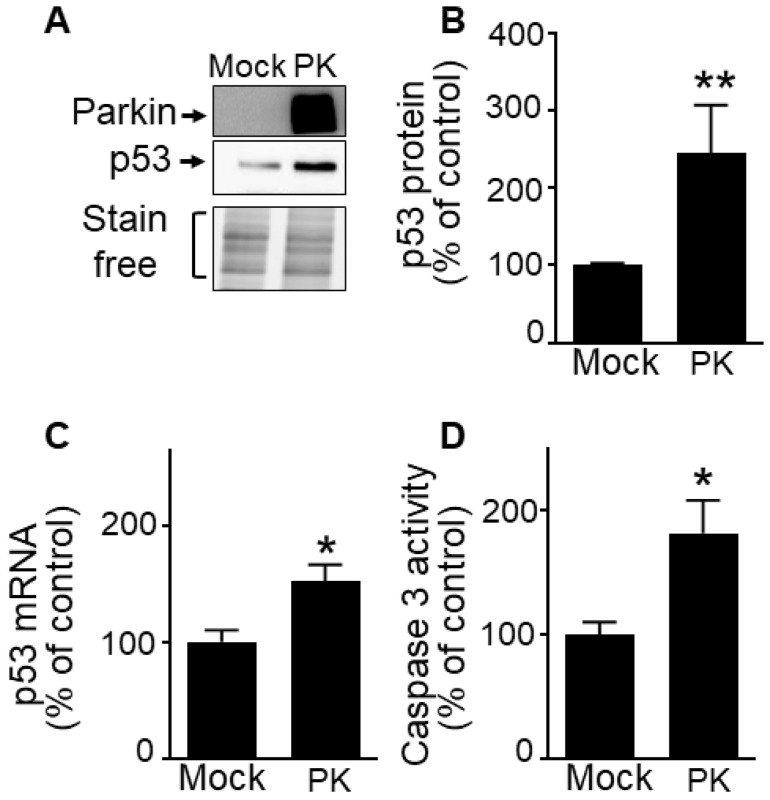
** PK up-regulates p53 in GBM cells. (A-D)** U87 cells stably overexpressing an empty vector (Mock) and wild-type PK (PK^WT^) obtained by transfection approach were examined for p53 expression (A, B, N = 9), *p53* mRNA levels (C, N = 6) and caspase 3 activity (D, N = 6) as described in Methods. Data are expressed as percent of Mock control cells (taken as 100%) and are the means +/- SEM of 2-4 independent experiments performed in triplicates. Stain free labeling is provided in (A) as a control of protein load. Statistical analysis was performed by Kruskal-Wallis test/Dunn's multiple comparison test. * *p* < 0.05; and ** *p* < 0.01.

Our previous work established that PK down-regulates p53, a master regulator of cell cycle 33-36 and apoptosis in post-mitotic neurons 12. Whether a similar phenotype could be observed in a GBM proliferating context remained to be established. Interestingly, we demonstrate that unlike observed in neurons, wild-type PK increases p53 protein (**Figure 6A, B**) and mRNA levels (**Figure 6C**) in U87 cells. Corroborating the pro-apoptotic role of PK via the up-regulation of p53, we have observed a PK-linked increase of caspase-3 activity in U87 GBM cells (**Figure 6D**).

In this context, we aimed at establishing whether PK-dependent control of cyclins could be also partly dependent of p53. Thus, we analyzed PK-linked cyclins regulation in 8MG cells, a GBM cell line in which p53 is biologically inactive (http://p53/free.frp53@free.fr). Supplementary **Figure** S2 shows that PK overexpression in these cells still leads to a repression of cyclins A and B protein (**Figure S2A, B**), promoter activity (**Figure S2C, D**) and mRNA levels (**Figure S2E, F**) indicating that PK controls these genes independently of p53 and further corroborates our demonstration (**Figure 3A, B**) of a direct control of cyclins promoter repression by PK.

### PK depletion triggers increased brain tumor volume and enhances cyclins A and B expressions *in vivo*

**Figure 7:**
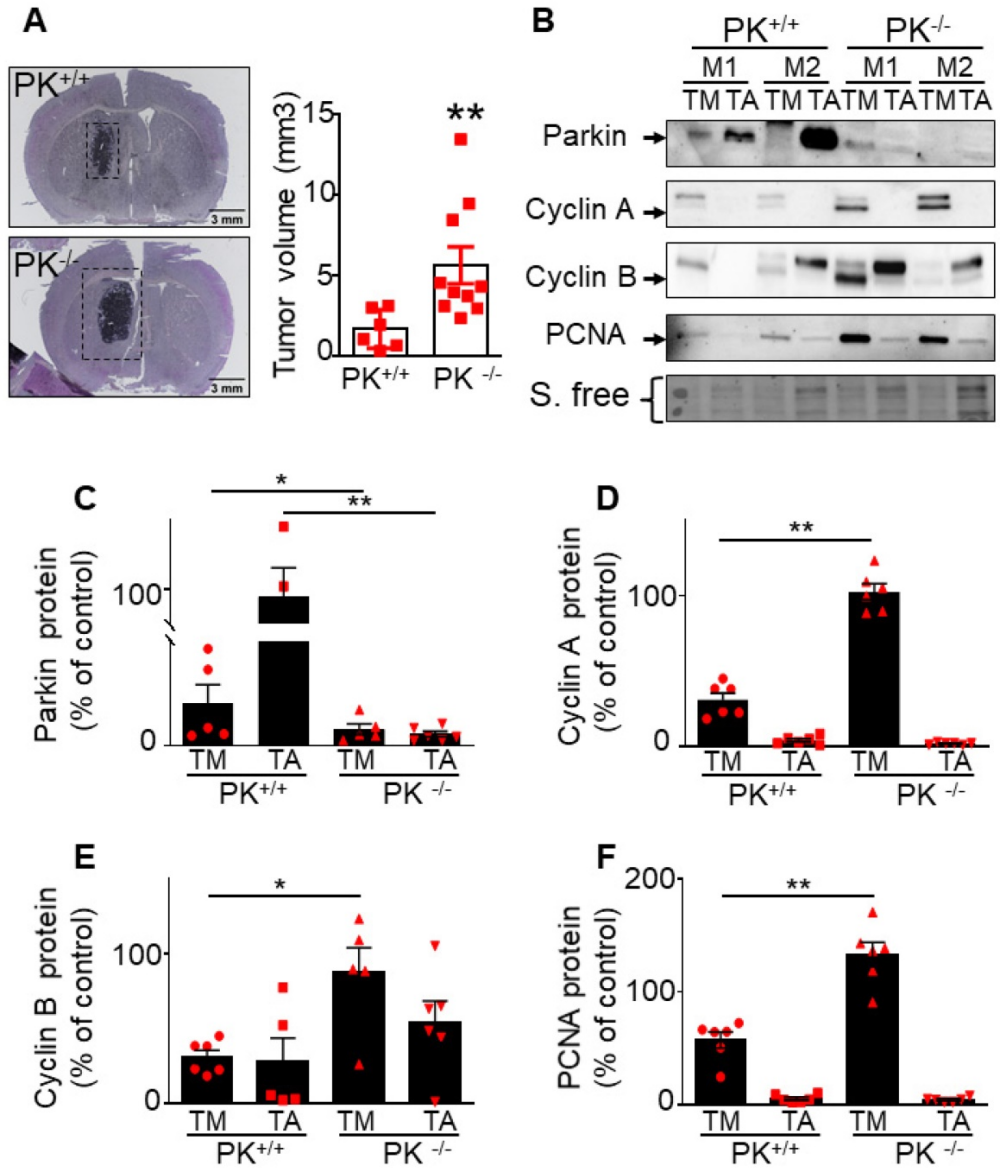
** Endogenous PK triggers tumor suppression *in vivo*. (A)** Analysis of tumor volume in control (PK^+/+^) and invalidated (PK^-/-^) mouse (M) brains injected with GL261 GBM cells as described in Methods. Results are expressed as the means +/- SEM of N = 6-10 mice. Statistical analysis was performed by Mann-Whitney test. **(B-F)** Analysis of PK (B, C, N = 6), cyclin A (B, D, N = 6), cyclin B (B, E, N = 6) and PCNA (B, F, N = 6) protein expressions in the tumor (TM) and adjacent tissue (TA) in PK^+/+^ and PK^-/-^ mice brains. Data are expressed as percent of PK^+/+^ TM expression (taken as 100%). Stain free (S. free) labeling is provided in (A) as a control of protein load. Statistical analysis was performed by Mann-Whitney test. * *p* < 0.05 and ** *p* < 0.01.

**Figure 8:**
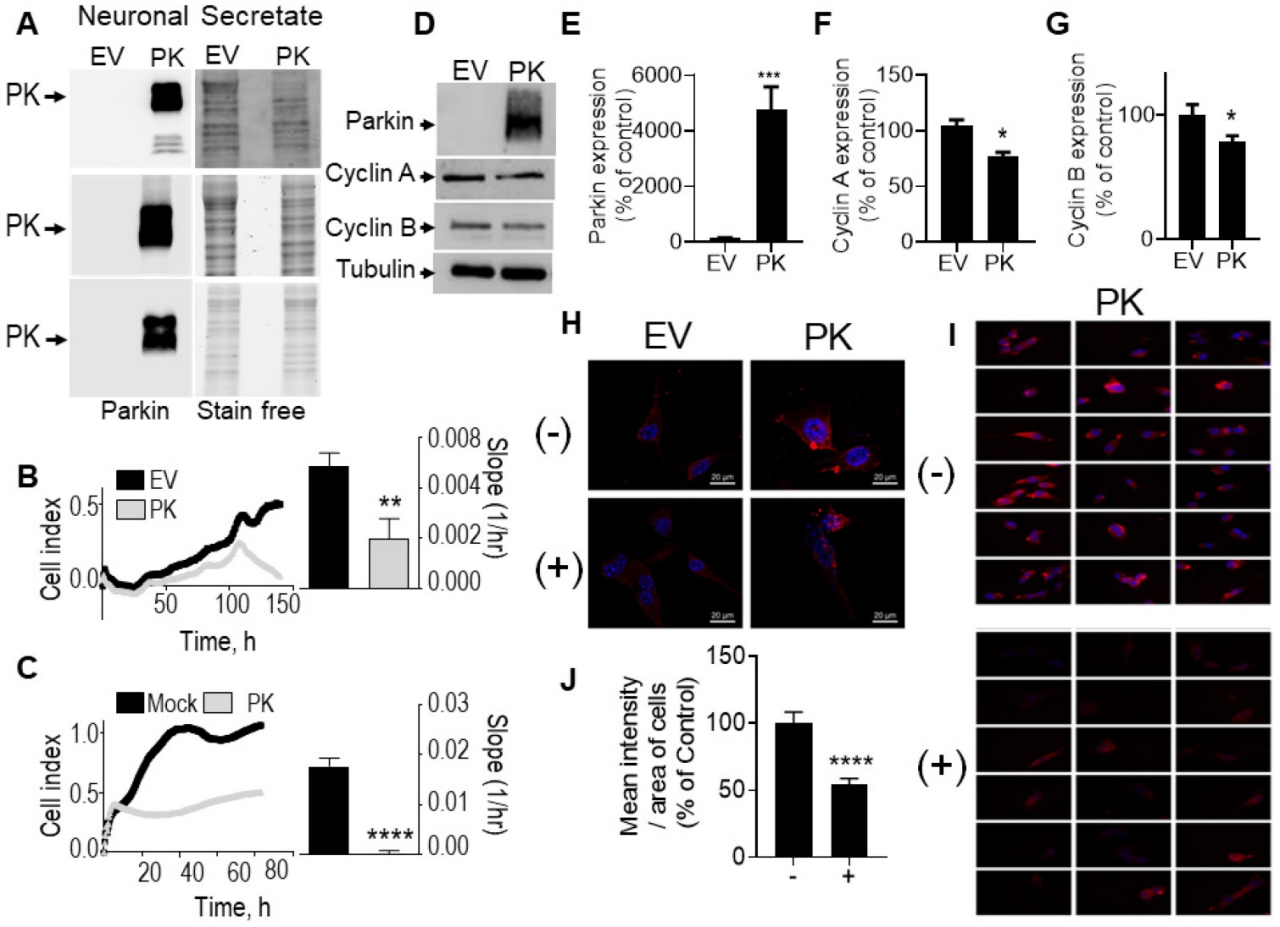
** PK-containing neuronal secretates influence glioblastoma cell proliferation, cell cycle and cyclins expression. (A)** PK expression in secretates of EV- or PK^wt^ -over-expressing TSM1 cells (3 independent experiments). Stain free signals serve as loading controls. **(B)** Impact of SH-SY5Y cells over-expressing PK or an empty vector (EV) on U87 cells in co-culture experiments as described in Methods. Quantification analysis (slopes) correspond to the means +/- SEM of 2-3 independent experiments performed in triplicates. **(C)** Impact of secretates from mock-transfected or PK-expressing TSM1 cells on GL261 proliferation evaluated with the xCELLigence technology as described in Methods. Quantification analysis of the slopes corresponds to the means +/- SEM of 2 independent experiments performed in quadruplicates. **(D-G)** Analysis of PK (D, E, N = 9), cyclin A (D, F, N = 9) and cyclin B (D, G, N = 6) protein expression in GL261 cells treated for 24 hours with secretates produced from EV- or PK-expressing TSM1 neuronal cells. Data are expressed as percent of EV control secretate (EV) taken as 100%. Tubulin labeling is provided in (D) as a control of protein load. Statistical analysis was performed with Mann-Whitney test. * *p* < 0.05 and *** *p* < 0.01. **(H)** PK expression detected by immunofluorescence microscopy in GL261 cells treated for 20 hours with secretates (200 µg of proteins) prepared from EV- or PK^wt^ -over-expressing TSM1 cells with in absence (-) or in the presence (+) of Dynasore (40 µM). Images were obtained by epifluorescence and deconvolved as described in the methods. **(I)** Panels of eighteen images corresponding to GL261 cells incubated with the secretate of PK-expressing TSM1 cells in absence (-, N = 241) or in the presence (+, N = 140) of Dynasore. Images were obtained by epifluorescence as described in the methods. **(J)** Mean intensity of PK immunostaining per area of GL 261 cells treated for 20 hours with secretates of PK-expressing TSM1 cells, treated or not with Dynasore (40 µM). The means are calculated with the free software CellProfiler4. Data are expressed as percent of untreated cells. Statistical analysis was performed using an unpaired t test with Welch's correction, **** *p* < 0.001.

To evaluate whether endogenous PK could interfere with brain tumor development *in vivo,* we have stereotaxically injected naïve GL261 tumor cells in brain of control (PK^+/+^) and PK knockout mice (PK^-/-^). **Figure 7A** clearly shows that *PRKN* gene invalidation leads to increased tumor volume (see representative coronal sections) corroborating the tumor suppression properties of endogenous PK. Importantly it also suggests that PK modulation in tumor environment impacts tumor development. This observation led us to examine the possible molecular mechanisms underlying this phenotype. A possibility could be that PK could be secreted by neuronal cells, captured by GL261 tumor cells and, once inside GL261 cells could regulate cyclins protein levels. To test this hypothesis, we have first analyzed the conditioned media (CM) of neuronal cells overexpressing PK. As shown in **Figure 8A,** high levels of PK are readily detectable in the secretates of PK-expressing TSM1 neurons. Since quantification analysis of PK was not possible due to poorly detectable expression of PK in controls cells (EV), we have provided a representative gel of three independent experiments as well as stain free images attesting equal protein charge. Then, we have analyzed if secreted PK could modulate GL261 cells proliferation by XCelligence approach. **Figure 8C** shows that GL261 cells treated with the CM of PK-expressing TSM1 neurons proliferate slower (compare black versus gray curves and quantification analysis of the slopes) than GL261 cells treated with the CM of EV-transfected TSM1 neurons. Next, we have examined if the decreased proliferation of GL261 by the conditioned media of TSM1 overexpressing PK was associated to a recapture of exogenous PK and consequent regulation of cyclins A and B. **Figure 8D, E** shows that GL261 cells display undetectable intracellular levels of PK when treated with the conditioned media of control neurons while a drastic increase of PK expression was observed upon treatment with the conditioned media of PK overexpressing TSM1 neurons. This biochemical data was confirmed *in situ*, by epifluorescence microscopy analysis (**Figure 8H, I**). Interestingly, PK recapture by GL261 cells was prevented by the dynamin inhibitor dynasore (**Figure 8H-J**). This data demonstrates that secreted PK may be recaptured by tumor cells. Further, **Figure 8F, G** indicates that recaptured PK leads to decreased levels of cyclin A (**Figure 8F**) and cyclin B (**Figure 8G**), in agreement with the decrease of proliferation described in **Figure 8C**. It should be noted that U87 cells co-cultured with SH-SY5Y cells overexpressing PK also show decreased proliferation (see quantification of curves slopes) compared to U87 cells co-cultured with EV-transfected SH-SY5Y cells (**Figure 8B**), indicating that the above-described mechanism evidenced with murine cellular models that could account for the control of tumor in mice also occurs in a human cellular context.

Importantly this data is corroborated by *in vivo* analysis. Thus, after tumor induction by GL261 cells (that do not express PK), we have observed a higher PK expression (**Figure 7C**) in tumor punches (TM) from PK+/+ mice than in PK-/- mice, indicating a recapture of PK by tumor cells. As expected, adjacent tissue (TA) samples show high expression of PK in PK^+/+^ but not PK^-/-^ mice brains. Analysis of cyclin A (**Figure 7B, D**), cyclin B (**Figure 7B, E**) and PCNA (an additional classical marker of proliferation, **Figure 7B, F**) expressions in the tumor punches (TM) from PK^+/+^ and PK^-/-^ mice shows an accumulation of these proteins in PK^-/-^ TM punches when compared to PK^+/+^. Overall, this set of data provides a mechanistic insight of how PK-enriched tumor environment could block tumor development.

Finally, to test the therapeutic potential of PK *in vivo,* we have orthotopically injected control or PK over-expressing GL261 cells (see PK expression in **Figure S3A**) in PK^-/-^ mice brains. **Figure S3B** shows that the over-expression of PK lowers GL261-induced tumor development thus further strengthening the conclusion of a potent and efficient tumor suppressor function of PK in an orthotopic model of GBM.

### Expressions of PK and cyclins are correlated in human GBM biopsies

**Figure 9:**
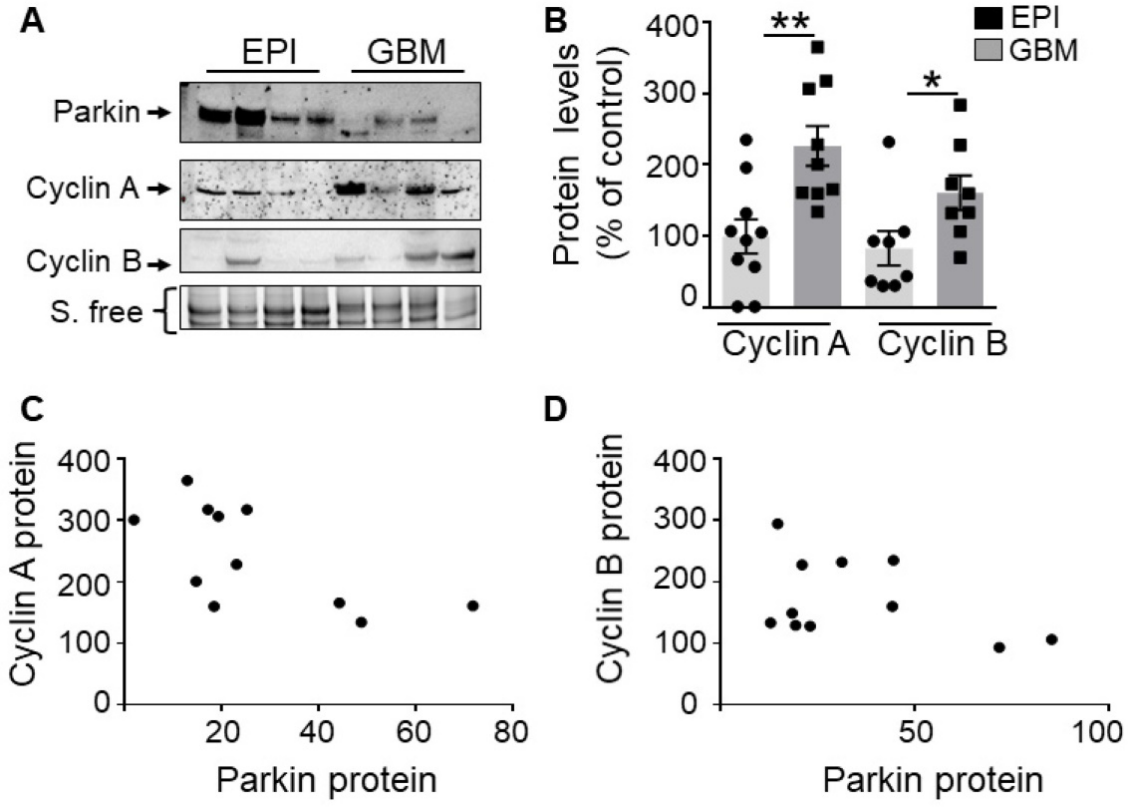
** PK and cyclins expressions in human GBM biopsies. (A, B)** Western-blot analysis of PK and cyclin protein levels in control (EPI,) and GBM samples as described in Methods. Data are expressed as percent of EPI cohort and are the means +/- SEM of N = 8-10 (EPI) and N = 8-9 (GBM) samples. * *p* < 0.05; ** *p* < 0.01. **(C, D)** Correlation analysis between PK and cyclin A (C, Spearman r 0.76, *p* < 0.05) or PK and cyclin B (D, Pearson r 0.78, *p* < 0.05) protein levels in GBM samples. Data in both axes are expressed as percent of control.

We have measured the levels of cyclins A, B and PK in human glioblastoma biopsies already characterized in 9. First, as previously described 9, we confirm a lower PK expression in GBM samples (**Figure 9A**) than in controls (EPI, epilepsy-derived samples corresponding to a non-correlated pathology). Second, we show that the proteins levels of cyclins A and B are significantly increased in GBM human samples (**Figure 9B**). Of most importance, r correlation analysis indicates a strong and significant inverse relationship between PK and cyclin A (**Figure 9C,** Spearman r of - 0.76, *P* = 0.036) or cyclin B (**Figure 9D,** Pearson r of - 0.78, *P* = 0.036) protein expressions.

## Discussion

Brain tumor development is linked to the deregulation of various processes governing cell transformation, but the alteration of cell cycle regulatory genes has evolved as a key molecular trigger of astrocytic gliomas 1, 37. It has been shown that PK reduces cell proliferation by blocking cell entry in the G1/S phase of the cell cycle via its E3-ligase activity. Hence, it has been shown that PK is involved in the ubiquitination and degradation of cyclins E and D, two regulators of CDK2 and CDK4/6, respectively 8, 10, 38. Our study is the first demonstration of a direct contribution of the transcriptional factor function of PK to the control of the S and G2/M phases of the cell cycle and that this mostly occurs through the transcriptional control of *cyclins A* and *B* genes, two key regulators of the S (synthesis) and G2/M (mitosis) phases of the cell cycle. Our demonstration stands on five independent lines of evidence. First, PK overexpression reduces *cyclins A* and *B* mRNA levels and promoter activities. Second, depletion of endogenous PK increases *cyclins A* and *B* mRNA and promoter activities. Third, PK-mediated repression of *cyclin A* and *B* promoter activities is abolished by removal of a PK-responsive element. Fourth, PK physically interacts with the promoters of cyclin A and B. Fifth, PK-induced repression of cyclins promoter activity and mRNA levels is not affected by p53 depletion. The trans-repression of *cyclins A* and *B* promoters elicited by PK *in vitro* and *in vivo* supports its well-established tumor suppressor role in GBM 8. These direct lines of evidence agree well with our observation of a drastic reduction of PK expression accompanied by an increase of cyclins levels in human patient's biopsies and the inverse relationship between PK and cyclins expressions in GBM biopsies (this work **Figure 9** and 39-41). Our data indicate a functional interaction between PK and cyclins *in vitro*, *in vivo* and also in human biopsies.

Interestingly, we have observed that E3-ligase and TF functions of PK differentially contribute to cyclins regulation. Thus, PK targeting to the cytosol compartment (PK^NESt^) abolishes cyclin A regulation while cyclin B modulation of expression is not only preserved in PK^NESt^ expressing cells (**Figure 5**) but even further reduced due to enhanced E3-ligase activity in PK-enriched cytosol. Such E3-ligase-mediated PK-mediated regulation of cyclin B has been previously documented and accounted for by the ability of PK to interact with the co-activators of the anaphase-promoting complex/cyclosome (APC/C), Cdc20 and Cdh1^42^. This agrees with our study, which represents the first demonstration of a transcriptional PK-mediated control of cyclins A and B and a dual E3-ligase and TF PK contribution to cyclin B regulation.

Our study may have general implications for future studies aimed at delineating the respective contribution of PK ligase and TF functions. Our study is an example of such possibility since even if probably both activities contribute to tumor suppression, nuclear and cytosolic PK may distinctly contribute to the regulation of key cell cycle players, suggesting that both PK functions should be examined and targeted to maximize its therapeutic potential. In this context, it should be emphasized that several mutations have been shown to abolish PK ubiquitin ligase function. However, their putative influence on PK TF function had not been established. Our study shows that one of these mutations, C431S, that constitutes the key catalytic residue of PK E3-ligase activity, could also well affect PK TF function as it drastically alters not only protein expressions but also mRNA levels of cyclins. However, it cannot be excluded that the impairment of the E3-ligase activity of PK by the C431S mutation could modulate the protein levels of additional transcription factors involved, besides PK, in cyclins regulation.

Thus, the study of PK mutants allowing selective sequestration of PK in cytosolic or nuclear compartments should prove useful for decrypting the specific transcription factor and E3-ligase molecular transcriptional and post-transcriptional interactomes and thus, improve the development of therapies targeting either PK TF or E3-ligase functions. Moreover, considering that PK mis-localization may deeply impact its function, it will be important to determine if the PK shuttling between nucleus and cytosol is affected during GBM setting and/or progression and if the cellular context drives PK-linked alterations in GBM.

Importantly we have shown that PK knockout mice show increased tumor volume indicating that a cellular environment depleted in PK may negatively impact GBM tumor progression *in vivo* (**Figure 7**). In this context, we postulated that PK expression of cells surrounding tumors may control its progression by exocrine production of tumor suppressor effectors and envisioned that PK itself could well act as such. This hypothesis supposes that PK could be released from surrounding cells and act, at distance after its recapture by target tumor cells. It happened that this postulate was exact. Thus, we have demonstrated that PK can be secreted by neuronal cells and recaptured in a dynamin-dependent manner, by tumor cells. Importantly, after recapture by tumor cells, PK reduces cellular proliferation in host cells via cyclins A and B regulation both *in vitro* and *in vivo* (**Figures 7 and 8**). Further studies will be necessary to establish the beneficial impact of a PK- “enriched” cellular environment for GBM treatment. Accordingly, the delineation of the mechanisms underlying the cellular interaction between neurons and other brain cell types that are “enriched” in PK and glial cells may unravel the putative role of PK in additional processes involved in cell transformation and GBM development.

Finally, we have also shown that in U87 GBM cells, PK up-regulates the transcription of p53 (**Figure 6**), a master regulator of cell cycle and apoptosis 33-36. These results provide the first experimental data that may explain the apparently opposite phenotypes elicited by PK in post-mitotic neurons versus proliferating glioblastoma cells. Thus, we have previously shown that, in SH-SY5Y dopaminergic neurons, PK induces neuroprotection via the transcriptional repression of p53 12. This apparent opposite and cell type-specific phenotype elicited by PK is not unusual and reported for various transcriptional factors 43.

Since p53 may also negatively regulate cyclins A and B 44, 45, we assessed whether PK could also control cyclins indirectly via p53. We show that PK still regulates cyclins transcription in 8MG (**Figure S2**), a glioblastoma cell line in which p53 is functionally inactivated by mutation, indicating that PK-mediated control of cyclins A and B is fully p53-independent in glioblastoma cells. Whether the pro-apoptotic effect of PK is dependent of the mutational status of p53 and if PK E3-ligase contributes to the elimination of aggregated p53 remains to be established.

Overall, our work highlights the importance of the transcriptional function of PK in gliomagenesis. Thus, we have identified two novel PK transcriptional targets (cyclins A and B) and demonstrated that PK TF function can work in synergy with its E3-ligase activity or independently, depending on the cyclin examined. Moreover, we show that the control of PK targets is dependent of the cellular context and thus explain the apparent paradoxical observation of a dual ability to induce either neuroprotection or tumor suppression. Finally, we show for the first time that PK can be secreted from neurons and act at distance as a protective factor slowing down the progression of cerebral tumors.

## Supplementary Material

Supplementary figures and tables.

## Abbreviations

BSA: bovine serum albumin
CDK: cyclin dependent kinase
ChIP: chromatin immuno precipitation
Ct: control
DAPI: 4',6-diamidino-2-phénylindole
DMEM: Dulbecco's modified Eagle's medium
DMS: donkey mouse
DNA: deoxyribonucleic acid
EGFP: enhanced green fluorescent protein
EV: empty vector
FACS: fluorescent activating cell sorting
GBM: glioblastoma
HAP1: human haploid cell line
IMDM: Iscove's modified Dulbecco's medium
MEF: mouse embryonic fibroblasts
mRNA: messenger ribonucleic acid
NEO: neomycin
NES: nuclear export signal
NH4Cl: ammonium chloride
OME: open microscopy environment
PBS: phosphate-buffer saline
PCR: polymerase chain reaction
PK: parkin
PKRE: parkin responsive element
qPCR: quantitative polymerase chain reaction
RNA: ribonucleic acid
rpm: rotation per minute
RTCA: real time cell analysis
SEM: standard error of the mean
SD: standard deviation
SDS-PAGE: sodium dodecyl sulfate polyacrylamide gel electrophoresis
TF: transcription factor
