# Supplementary material for: Therapeutic potential of parkin as a tumor suppressor via transcriptional control of cyclins in glioblastoma cell and animal models
Source: Theranostics. 2021 Nov 1;11(20):10047–63. doi: 10.7150/thno.57549 (PMC8581414; doi:10.7150/thno.57549)
Supplement: Supplementary file 1 — Supplementary figures and tables. [file thnov11p10047s1.pdf]

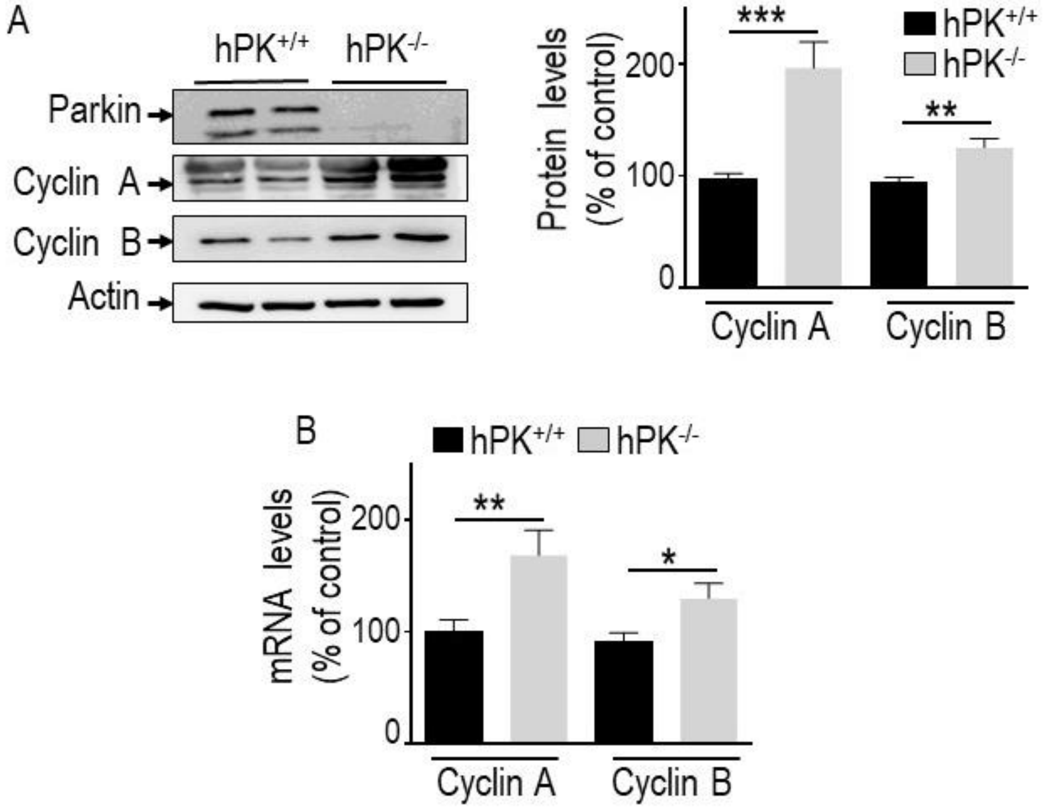

Figure S1. Endogenous human PK controls cyclins protein and mRNA levels.

(A, B) HAP control ($hPK^{+/+}$) or invalidated for PK ($hPK^{-/-}$) were assessed for cyclin A (A, N = 9, analyzed by Student's test) and cyclin B (A, N = 6, analyzed by Mann-Whitney test) protein expressions and cyclin A (B, N = 15, Student's test) and cyclin B (B, N = 12, Mann-Whitney test) mRNA levels as described in Methods. Data are expressed as percent of $hPK^{+/+}$ control cells (taken as 100%) and are the means +/- SEM of 2-5 independent experiments performed in triplicates. Actin expression is provided in (A) as a control of protein load. * $p < 0.05$; ** $p < 0.01$; *** $p < 0.001$ and ns = non significant.

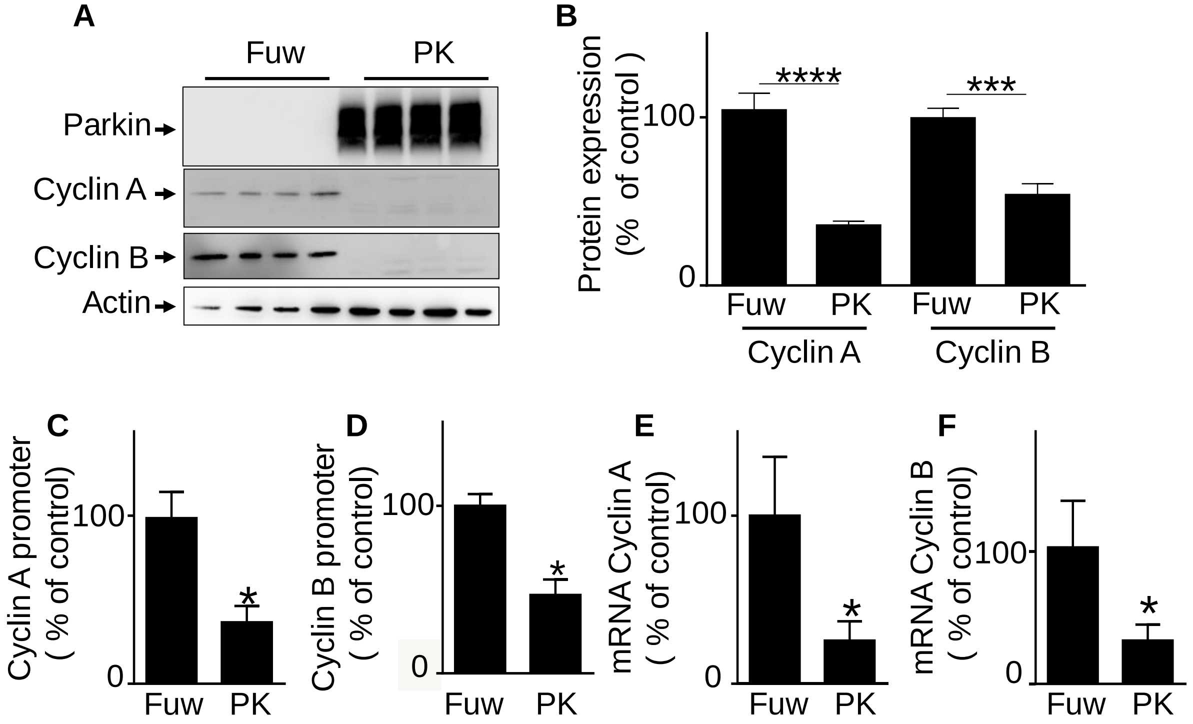

Figure S2. PK-mediated regulation of cyclins in 8MG cells.

(A-F) 8MG cells stably expressing either an empty Fuw lentiviral vector or wild-type PK were assessed for cyclin A and B (A, B, N = 9, student t test) protein expressions; cyclin A (C, N = 6, Mann-Whitney test) and cyclin B (D, N = 6, Mann-Whitney test) promoter activity and cyclins A (E, N = 6, Mann-Whitney test) and B (F, N = 6, Mann-Whitney test) mRNA levels as described in Methods. Data are expressed as percent of Fuw control cells (taken as 100%) and are the means +/- SEM of 2-3 independent experiments performed in triplicates. Actin is provided in (A) as a control of protein load. * $p < 0.05$; *** $p < 0.001$; **** $p < 0.0001$.

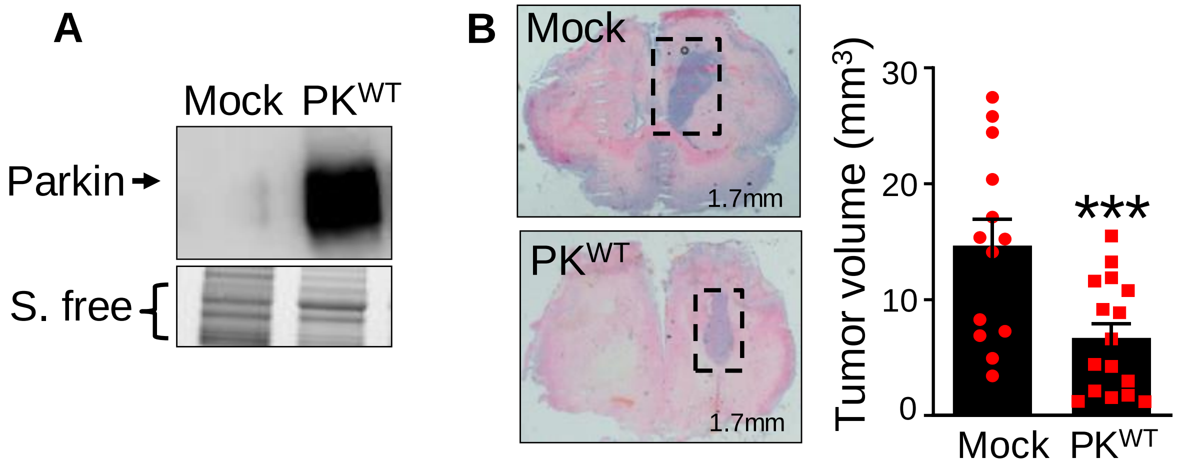

Figure S3. PK overexpression prevents tumor progression *in vivo*.

(A) GL261 glioblastoma cells stably overexpressing an empty vector (Mock) and wild-type PK (PK^{WT}) were examined for PK expression as described in Methods. The stain free panel serves as loading control.

(B) Analysis of tumor volume in brains of mice injected with either Mock- or PK^{wt}-transfected GL261 GBM cells. Representative brain sections colored with Hematoxylin-Eosin to visualize and locate the tumor are shown for Mock and PK^{wt} conditions. Tumor volume was measured as described in Methods. Data are the means +/- SEM (Mock N = 13, PK^{wt} N = 16, Mann-Whitney test).
